# Maternal Obesity Caused by Overnutrition Exposure Leads to Reversal Learning Deficits and Striatal Disturbance in Rats

**DOI:** 10.1371/journal.pone.0078876

**Published:** 2013-11-04

**Authors:** Ting Wu, Shining Deng, Wei-Guang Li, Yongguo Yu, Fei Li, Meng Mao

**Affiliations:** 1 West China Second Hospital, Sichuan University, Chengdu, Sichuan, China; 2 Department of Developmental and Behavioral Pediatrics, Shanghai Institute of Pediatric Translational Medicine, Shanghai Children's Medical Center, Shanghai Jiao Tong University School of Medicine, Shanghai, China; 3 Chengdu Women's and Children's Central Hospital, Chengdu, Sichuan, China; 4 MOE-Shanghai Key Laboratory of Children's Environmental Health, Shanghai Jiao Tong University School of Medicine, Shanghai, China; 5 Departments of Anatomy and Embryology, Biochemistry and Molecular Cell Biology, Shanghai Key Laboratory for Tumor Microenvironment and Inflammation, Institute of Medical Sciences, Shanghai Jiao Tong University School of Medicine, Shanghai, China; University of Santiago de Compostela School of Medicine – CIMUS, Spain

## Abstract

Maternal obesity caused by overnutrition during pregnancy increases susceptibility to metabolic risks in adulthood, such as obesity, insulin resistance, and type 2 diabetes; however, whether and how it affects the cognitive system associated with the brain remains elusive. Here, we report that pregnant obesity induced by exposure to excessive high fatty or highly palatable food specifically impaired reversal learning, a kind of adaptive behavior, while leaving serum metabolic metrics intact in the offspring of rats, suggesting a much earlier functional and structural defects possibly occurred in the central nervous system than in the metabolic system in the offspring born in unfavorable intrauterine nutritional environment. Mechanically, we found that above mentioned cognitive inflexibility might be associated with significant striatal disturbance including impaired dopamine homeostasis and disrupted leptin signaling in the adult offspring. These collective data add a novel perspective of understanding the adverse postnatal sequelae in central nervous system induced by developmental programming and the related molecular mechanism through which priming of risk for developmental disorders may occur during early life.

## Introduction

Current data from epidemiological as well as *in vivo* animal studies have contributed to the concept of developmental programming, whereby an unfavorable prenatal environment is believed to be associated with long-term metabolic consequences [1], [2]. A recent longitudinal study revealed that maternal obesity not only predisposed the fetus to develop obesity, insulin resistance and type 2 diabetes [3], but also adversely affected IQ performance, emotional problems and core symptoms of attention deficit and hyperactivity disorder (ADHD) in school-aged children [4]–[6]. Although the postnatal sequelae in the central nervous system induced by development programming has been noticed, current literature [4]–[6] based on the human studies are limited and inconsistent, from which it is difficult to accurately identify the etiology and related mechanisms linking maternal obesity to offspring's cognition, thus clinically posing obstacle in guiding the pregnant woman to achieve a healthy weight. Besides, the adverse neurodevelopmental outcomes in offspring could be, at least partly, caused by the predisposed overweight that usually develops later in the offspring's life [3]. Therefore, whether and how maternal obesity directly affects the cognitive system remains to be established.

Cognitive flexibility is an essential ability of adaptive behaviors while cognitive rigidity is a behavioral symptom related to impulsivity and compulsivity, which is often detected in many developmental disorders, including ADHD, autism, Torette syndrome, Rett syndrome and schizophrenia [7]–[10]. As a kind of behavioral paradigm to evaluate the cognitive flexibility, reversal learning is defined as the ability to modulate responses to a previously non-reinforced stimulus, which indexes vulnerability for disorders characterized by impulsivity such as proclivity for initial substance abuse and compulsive aspects for the dependence [11]. In contrast to reversal learning which pertains to a specific exemplar, attentional set-shifting, another paradigm of cognitive flexibility, refers to the ability to adapt behavior flexibly following feedback, but pertains to broader stimulus dimensions (e.g. from lines to shapes, from texture to odor) [12]. Recently, it has been reported that core symptoms of ADHD, such as attention deficit and hyperactivity, are readily detected in the children born to the obese mothers [3], suggesting a stunted cognitive flexibility including reversal learning and attentional set-shifting likely associated with maternal obesity, since these disrupted behaviors are frequently co-developed in the ADHD patients through some shared mechanisms, although the direct causality and the underlying mechanisms still remain to be examined.

The present study was designed to investigate the impact of maternal obesity caused by overnutrition pre-conception and during pregnancy on the abilities of reversal learning and attentional set-shifting in rats and determine the potential mechanisms underlying the bio-communication between mother and offspring.

## Materials and Methods

### Ethics statement

All experiments of research were approved by the Animal Ethics Committee of Shanghai Jiao Tong University School of Medicine, China.

### Diets

The commercial chow diets were purchased from Research Diets Company, USA. The standard control (Ctrl) diet (D10012G) contained 64% carbohydrate, 20% protein and 7.0% fat; high-fat (HF) diet (D12492) 26.3% carbohydrate, 26.2% protein and 34.9% fat; highly palatable (HP) diet (D12329) 74.3% carbohydrate, 16.8% protein and 4.8% fat. The energy content (Kcal/100 g of dry diet, Kcal%) is 390 Kcal% for Ctrl diet, 524 Kcal% for HF diet and 407 Kcal% for HP diet. In Ctrl diet, 63.9 Kcal% of energy was distributed in carbohydrate, 20.3 Kcal% in protein and 15.8 Kcal% in fat; in HF diet, 20.0 Kcal% in carbohydrate, 20.0 Kcal% in protein and 60.0 Kcal% in fat; in HP diet, 73.1 Kcal% in carbohydrate, 16.4 Kcal% in protein and 10.5 Kcal% in fat.

### Care and maintenance of animals

Wistar rats were purchased from Shanghai Laboratory Animal Center, and maintained under controlled temperature with food and water provided *ad libitum*. Thirty 9-week-old female and 10 male were housed in individual cages under controlled temperature at 22°C, with a 12:12-h light-dark cycle (light on 7:00 AM), and were given commercial standard pellets for 1 week for adaption. Three groups of females were then formed according to the Ctrl, HF or HP diet (n = 10 in each group). After 6 weeks, females were caged in collective cages for mating and returned into individual cages after 14 days. At the time of birth, the male pups were kept for further investigation. After weaning, three groups of male pups were fed on Ctrl diet and allowed to free access to food and water. For the subject representativeness as well as devoid of behavioral variability, one to two male offsprings with the median weight(s) among the littermates from each mother (n = 9 for Ctrl group, n = 10 for HF group and n = 11 for HP group) were picked up for the further investigation on the following metabolic and behavioral examination.

### Experiment 1: Body weights of dams and offspring

**Figure 1:**
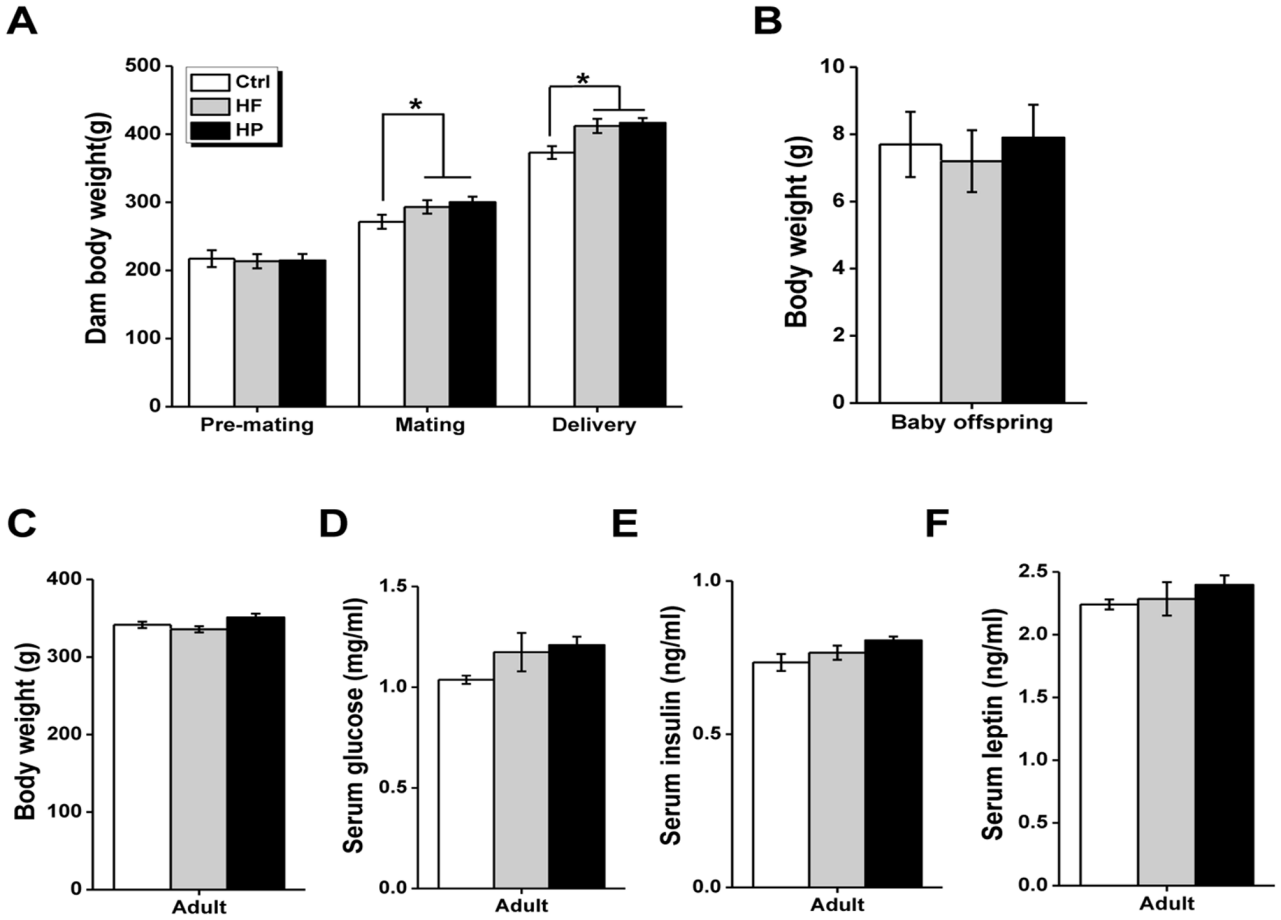
Effect of maternal diet on dam, baby and adult offspring. (A) Mean body weights of dam fed with the control diet (Ctrl), high fatty (HF) or highly palatable diet (HP) (n = 10 in each group). Dam were weighted 6 weeks before mating, at the time of mating and after the delivery of their offspring. (B–C) Mean body weight of offspring born to dams fed with the Ctrl diet (n = 9), HF diet (n = 10), and HP diet (n = 11), measured at birth (day 1), and in adulthood (10 weeks after birth). (D–F) Physiological parameters of offspring born to dams fed with the Ctrl diet (n = 9), HF diet (n = 10), and HP diet (n = 11). *p<0.05; **p<0.001.

Dams were measured at three time points (6 weeks before mating, at the time of mating and after the delivery of their offspring) (n = 10 in each group, Fig. 1A). In addition, the pups were weighted at birth and the age of 10 weeks (n = 9 for Ctrl group, n = 10 for HF group, and n = 11 for HP group, Fig. 1B–C).

### Experiment 2: Plasma parameter determination

The 10 weeks-old adult offspring (n = 9 for Ctrl group, n = 10 for HF group, and n = 11 for HP group, Fig. 1D–F) were fasted overnight and received intraperitoneal injection of recombinant rat leptin (1 mg/kg) (Huijia Technology, Xiamen, China, cat #: CYT-592) or saline. After 30 minutes, rats were killed by decapitation after cervical elongation. Blood was collected on heparin (10 IU/ml) and hypothalamus was quickly removed. The hypothalamus was immediately frozen into liquid nitrogen. Plasma glucose level was measured by enzymatic assay (Sigma-Aldrich, St. Louis, MO, USA). Insulin and leptin were assayed by radioimmunoassay using commercial diagnostic kits (LINCO Research, St. Louis, MO, USA). The other chemicals were purchased from Sigma-Aldrich (St. Louis, MO, USA).

### Experiment 3: Visual Discrimination and Serial Reversal Learning (VDSRL)

#### Apparatus

Operant conditioning chambers (30 cm ×24 cm ×30 cm; Med Associates, St Albans, VT, USA) were used to carry out behavioral tests, each chamber enclosed within a sound-attenuating wooden box which fitted with a fan for ventilation and masking of extraneous noise. In each chamber, two retractable levers were located on either side of a centrally positioned food magazine, into which an external pellet dispenser could deliver 45 mg sucrose pellets (Noyes dustless pellets; Sandown Scientific, Middlesex, UK). A light-emitting diode (LED) was positioned centrally above each lever. Besides, there were a magazine light and a house light. Magazine entry was detected by an infrared photocell beam located horizontally across the entrance. The software Med-PC-IV from Med Associates was used for apparatus control and behavioral recording.

#### Training and testing

**Figure 2:**
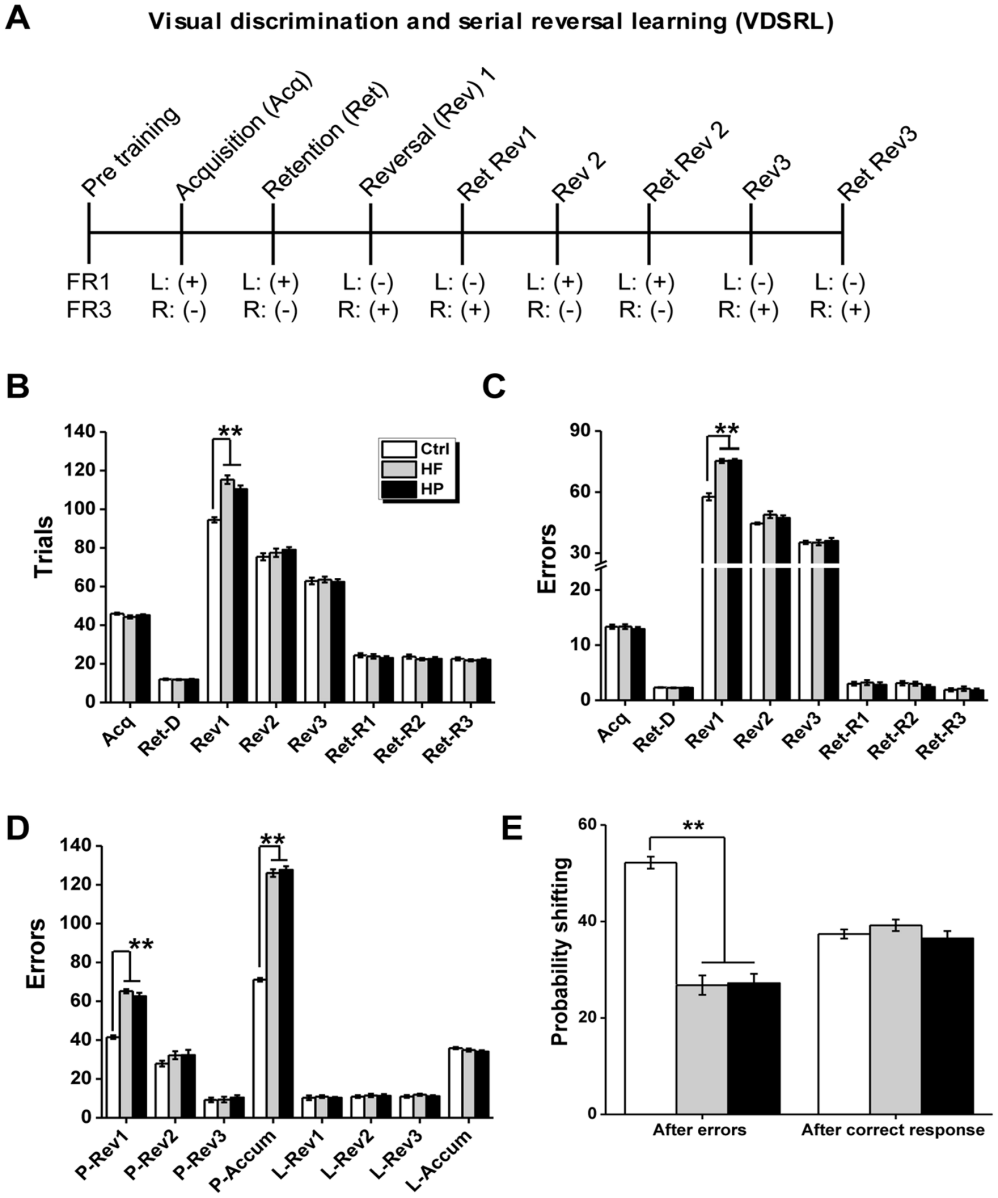
Reversal learning in the VDSRL task. (A) Schematic representation of the behavioral training and testing protocol. The rewarded and unrewarded lever is indicated by the “+” and “−”, respectively. The rewarded lever was counterbalanced across rats. (B–C) Total trials (B) and errors (C) to criterion during acquisition of spatial discrimination (Acq), retention of discrimination (Ret-D) as well as during the reversal phases (reversal 1–3: Rev1–3; retention of reversal 1–3: Ret-R1-3). (D) Perseverative and learning errors accumulated across reversals (perseverative errors accumulated: P-Accum;learning errors accumulated:L-Acuum) and during 3 reversals (perseverative errors of reversal 1–3: P-Ret1-3; learning errors of reversal 1–3: L-Ret1-3) were shown. A series of three reversals were performed by male offspring born to dams fed with the Ctrl diet, HF diet, and HP diet. Between successive reversals, animals were given a session to test retention of the previous reversal phase (Ret-R1-3). (E) Mean probabilities of rats shifting their responding to the other stimulus after making either an incorrect choice (and therefore not receiving reward) or a correct choice and receiving reward. n = 9 for Ctrl group, n = 10 for HF group and n = 11 for HP group. *p<0.05; **p<0.001.

A schematic representation of the behavioral training and testing protocol is shown in Fig. 2A, the protocol was adapted from Castañé *et al*. [13].

**Figure 3:**
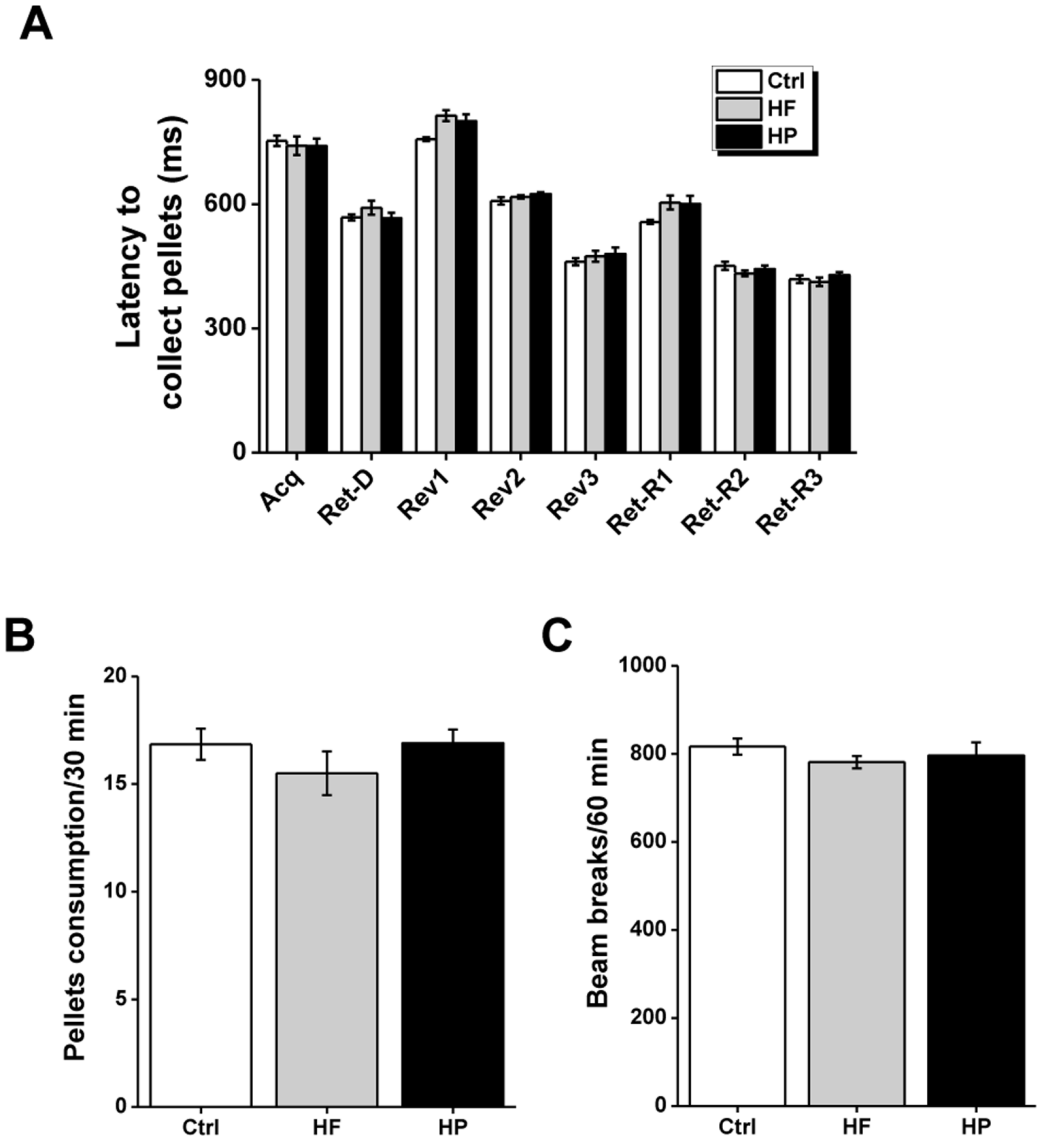
Feeding behaviors and locomotor activity of offspring in or after the VDSRL task. (A) Latencies to collect the food during acquisition (Acq), retention of spatial discrimination (Ret-D), reversal phase (Rev1-3), and retention of reversal 1–3 (Ret-R1-3) in the VDSRL task. A series of three reversals (reversal 1–3: Rev1–3) were performed. Between successive reversals, animals were given a session to test retention of the previous reversal phase (Ret-R1–3). *p<0.05; **p<0.001. (B) Food comsuption during a 30-min test session after the VDSRL task (n = 10 in each group). (C) Number of beam breaks during a 60-min test session after the VDSRL task (n = 9 for Ctrl group, n = 10 for HF group and n = 11 for HP group.).

Pre-training: All rats (n = 9 for Ctrl group, n = 10 for HF group, and n = 11 for HP group, Fig. 2A–C, Fig. 3A) were initially familiarized with the testing apparatus for 30 min. During this time, the house light was turned on and pellets were presented. After the habituation, training took place on each lever separately, firstly under a fixed ratio 1 (FR1) schedule to a criterion of 50 presses in 15 min, then under a fixed ratio 3 (FR3) schedule to a criterion of 150 presses in 15 min for each lever for 2 consecutive days. The FR3 schedule was used to avoid the possibility of reinforcing single, accidental presses on the correct lever. The presentation order of left and right lever was counterbalanced across subjects.

Acquisition of spatial discrimination: After the acquisition of a two-lever discrimination task as previously described [13], animals were trained to nose-poke the central magazine in order to trigger presentation of the retractable levers. Then, only three lever presses on one of these levers resulted in reward. Each session lasted 30 min and consisted of a maximum of seven 10-trial blocks. Each trial began with the switching on the house light. In the limited hold period of 20 s, the rat had to nose-poke in the magazine in order to provide the presentation of the two-levers which initiated a 10-s response interval. No response in either the limited hold period or the response interval resulted in the return to the inter-trial interval (ITI) state until the next trial was scheduled to begin, while the trial was recorded as an omission. Once the rat pressed one of the levers three times, both levers were retracted and the house light was turned off. Each rat had one training session per day and was trained until reaching a criterion of nine correct trials in one block of 10 trials (binomial distribution p<0.01, likelihood of attaining criterion in a 10-trial block). The completion of nine correct choices out of ten also implicated the end of the session. Once the criterion was reached, the initial discrimination phase was considered complete, and the animal was returned to the home cage. If the criterion was not achieved, this phase was repeated the next day till criterion achievement. Two days after acquisition, rats were retested on discrimination. Once the criterion was achieved, the reversal phase started.

Serial reversal learning task: During this phase, rats were trained to serially reverse an instrumental spatial discrimination in a between-session design. Three reversals were given, whereby in each reversal the previously correct lever became incorrect and the previously incorrect lever became correct (reversals 1–3). Each reversal session lasted 30 min and consisted of a maximum of seven 10-trial blocks. The learning criterion was the same as in the discrimination phase (nine correct trials in a 10-trial block). Animals usually required more than one session to reach criterion on the reversals. Thus, they received multiple, separate training sessions, the data of which were summed together. Between successive reversals, animals were given a session of up to seven 10-trial blocks where they were required to show retention of the previous reversal phase by reaching the nine of 10 correct criterion in one 10-trial block.

Behavioral measures: The main measures of the rat's ability to learn the discriminations/reversals were: (1) the number of trials to criterion and (2) the number of error to criterion (i.e. incorrect trials). Additional measures recorded for each trial were (3) the type of error, (4) the latency to collect the reward, and (5) the probability of shifting after an error or a correct response [14]. The type of errors was analyzed as described previously [15]–[17]. Briefly, sessions were classified as perseverative (where responding to the previously correct stimulus was significantly above chance), or learning (where responding to the newly correct stimulus was at, or above chance). The test was calculated by each rat's daily session considering the number of errors and the number of trials data. As an example, ≥44 errors in a 70 trials session indicated perseveration, between 43 and 27 errors was chance performance, and ≤26 errors showed accurate responding to the newly correct lever. Errors during perseverative sessions were considered perseverative errors and so on [15]–[17].

### Experiment 4: Food consumption

Food consumption of rats was tested as previously described [13]. Briefly, rats (n = 9 for Ctrl group, n = 10 for HF group, and n = 11 for HP group, Fig. 3B) were individually housed in standard rat cages where food pellets were freely available in a food cup. The amount of pellets consumed was calculated within 30 minutes.

### Experiment 5: Locomotor activity

Locomotor activity was measured by scoring beam breaks in activity chambers (Med Associates, St Albans, VT). A tracking system (Med Associates, St Albans, VT) was used to record activity during test sessions. Prior to open field tests, animals (n = 9 for Ctrl group, n = 10 for HF group, and n = 11 for HP group, Fig. 3C) were handled for two consecutive days. Standard rat cages were used as the novel open field.

### Experiment 6: Attentional set-shifting task (ASST)

#### Apparatus

**Figure 4:**
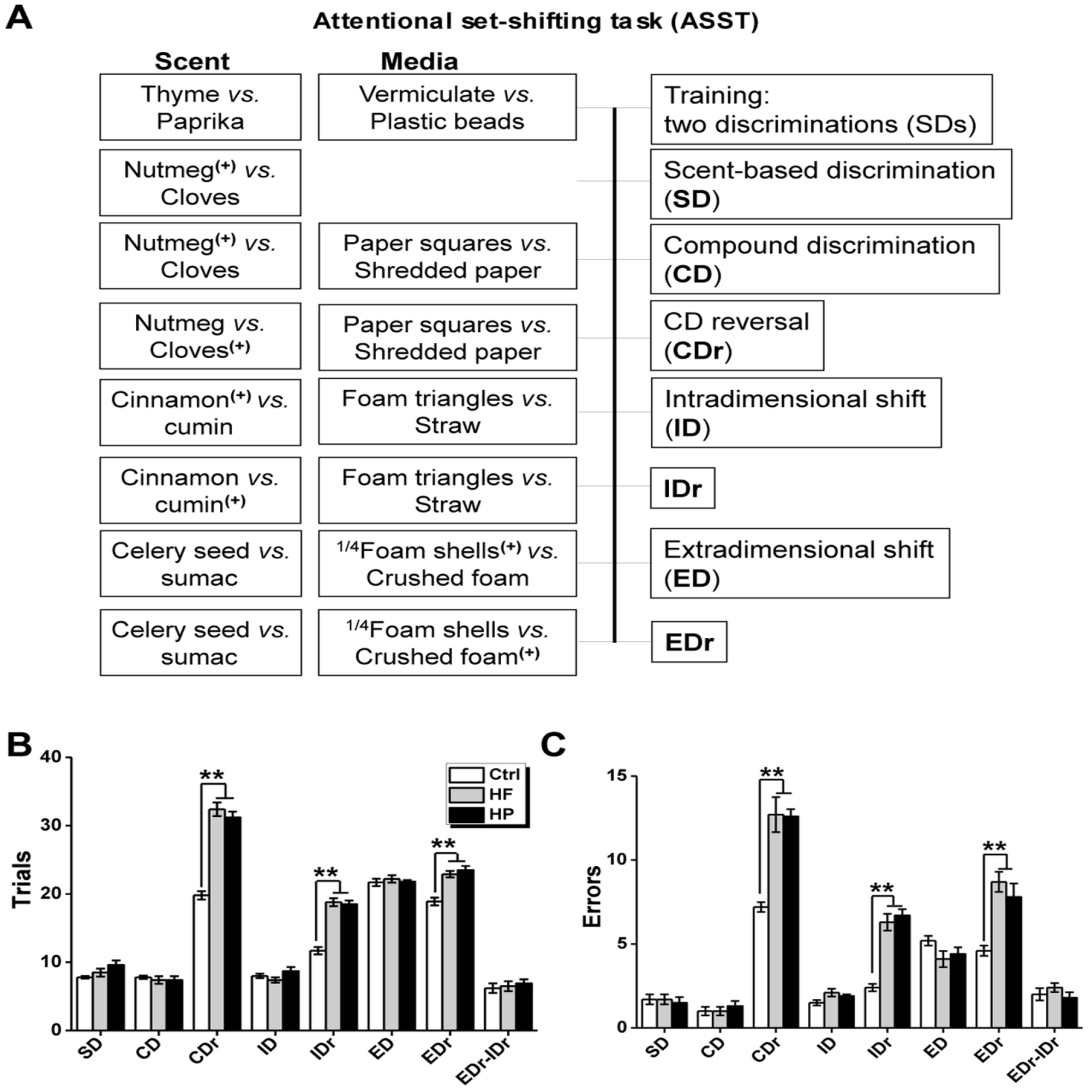
Reversal learning and set shifting in the ASST task. (A) Schematic representation of the behavioural training and testing protocol. The rewarded and unrewarded bowl is indicated with and without the “+”, respectively. The order of the discriminations and the exemplar pairings were always the same, but the pairs of exemplars were counterbalanced between groups. (B) Trials to reach criterion (six successive correct trials) for each phase of the ASST paradigm. (C) Errors made in each phase of the ASST paradigm. *p<0.05; **p<0.001.

Attentional set-shifting task (ASST) procedures were conducted according to Izquierdo et al. [18]. Rats (n = 9 for Ctrl group, n = 10 for HF group, and n = 11 for HP group, Fig. 4A–C) were trained in a Plexiglas arena that measured 36 cm (height) ×45 cm (width) ×68 cm (length). The box was divided equally into thirds so that each compartment was about 23 cm long. The front of the apparatus was further divided into two separate sections where the bowls were contained separately, to avoid animals having access to both bowls simultaneously. In addition, access to each compartment (and bowl) in the front of the box could be restricted by an opaque, removable divider. The back third of the apparatus (inter-trial chamber) was separated from the other compartments with a removable divider, and rats were placed in that chamber at the beginning of each trial. Access to the inter-trial chamber was blocked once a trial began.

Food rewards were buried half-way down ceramic bowls (4 cm tall, internal diameter is 8 cm). Rats were trained on successive days to make discriminations based on two dimensions: media of varying textures (e.g., vermiculite, confetti, gravel), or scents (e.g., paprika, thyme or oregano). Scents could be mixed interchangeably with media so that combinations of the two dimensions were possible, but pairs of scents or media were kept constant (e.g., cumin was always presented with cinnamon; vermiculite was always presented with gravel).

#### Training and testing

Food restriction and habituation: During food restriction, rats were fed about 13–17 g of food per day, and weight was monitored daily to maintain a target weight of 85% normal weight. To habituate the animals to the feeding bowls, and to familiarize them with digging for rewards, food was given in small ceramic bowls in the home-cage for several days before training. Rats were returned to free feeding once testing was complete, and they were kept on food deprivation for no more than 20 days. After deprivation, rats were habituated to the arena for 5 min before training. To familiarize the animals with digging for a food reward, animals were presented with two baited unscented bowls filled with home cage bedding. Once the animal had retrieved the food reward, the animal was moved back to the inter-trial chamber and the bowls were re-baited. The divider was then lifted, allowing the animal access to the bowls. All subsequent trials were conducted in this same manner. Once the animal was reliably digging for a food reward, training on the task was begun. A schematic representation of the behavioral training and testing protocol is shown in Fig. 4A, the protocol was modified as described previously [18].

Training: During the training phase, rats were trained on two simple discriminations (SDs), one scent-based (thyme vs paprika), and the second media-based (vermiculite vs plastic beads). All rats were trained on the same discriminations, in the same order. Criterion completion of training was achieved when the rat made a correct discrimination for six consecutive trials. Rats that did not learn to dig for food reward were returned to their home-cage and a second attempt was made one day later. More than 90% of rats completed the training within two attempts.

Testing: The day after training testing was delivered. A trial was initiated by raising the dividers, giving the rat access to two bowls, only one of which was baited. For each phase, the rat was given four discovery trials, whereby the rat was allowed to dig in both bowls to retrieve the food reward. Errors were recorded during the discovery trials, but did not count toward trials to criterion [18]. On subsequent trials, if the rat dug in the unbaited bowl, an error was recorded, and the trial was terminated. In a single-test session, rats were given the following discrimination phases to learn: (1) scent-based discrimination (SD, nutmeg^(+)^ vs cloves); (2) compound discrimination (CD), where media (paper squares vs shredded paper) dimension was introduced, while scent was still rewarded, irrespective of medium (nutmeg^(+)^/paper squares and nutmeg^(+)^/shredded paper); (3) CD-reversal (CDr), where previously unrewarded scent was now rewarded, irrespective of medium (cloves^(+)^/paper squares and cloves^(+)^/shredded paper); (4) intra-dimensional shift (ID), where animal must still attended to scent and correctly discriminated the rewarded scent, while novel scents (cinnamon^(+)^ vs cumin) and media (foam triangles vs straws) were introduced; (5) ID-reversal (IDr), where previously unrewarded scent was now rewarded, irrespective of medium (cumin^(+)^/foam triangles and cumin^(+)^/straws); (6) extra-dimensional shift (ED), where the rat was trained to attend to medium cues (1/4 foam shells^(+)^ vs crushed foam) and ignore scent cues (celery seed vs sumac); (7) ED-reversal (EDr), where previously unrewarded medium was now rewarded, irrespective of scent (crushed foam^(+)^/celery seed and crushed foam^(+)^/sumac). During each phase, the rats were tested until they had achieved a criterion of six consecutive correct choices. Two measures were used to quantify performance in each phase: (1) trials to criterion (the number of trials taken to reach criterion), and (2) the number of errors made in reaching criterion. The order of the discriminations and the exemplar pairings were always the same, but the pairs of exemplars were counterbalanced between groups. Typically, rats completed all phases of testing within a single day.

### Experiment 7: [^125^I]RTI-55 binding to dopamine transporter (DAT)

**Figure 5:**
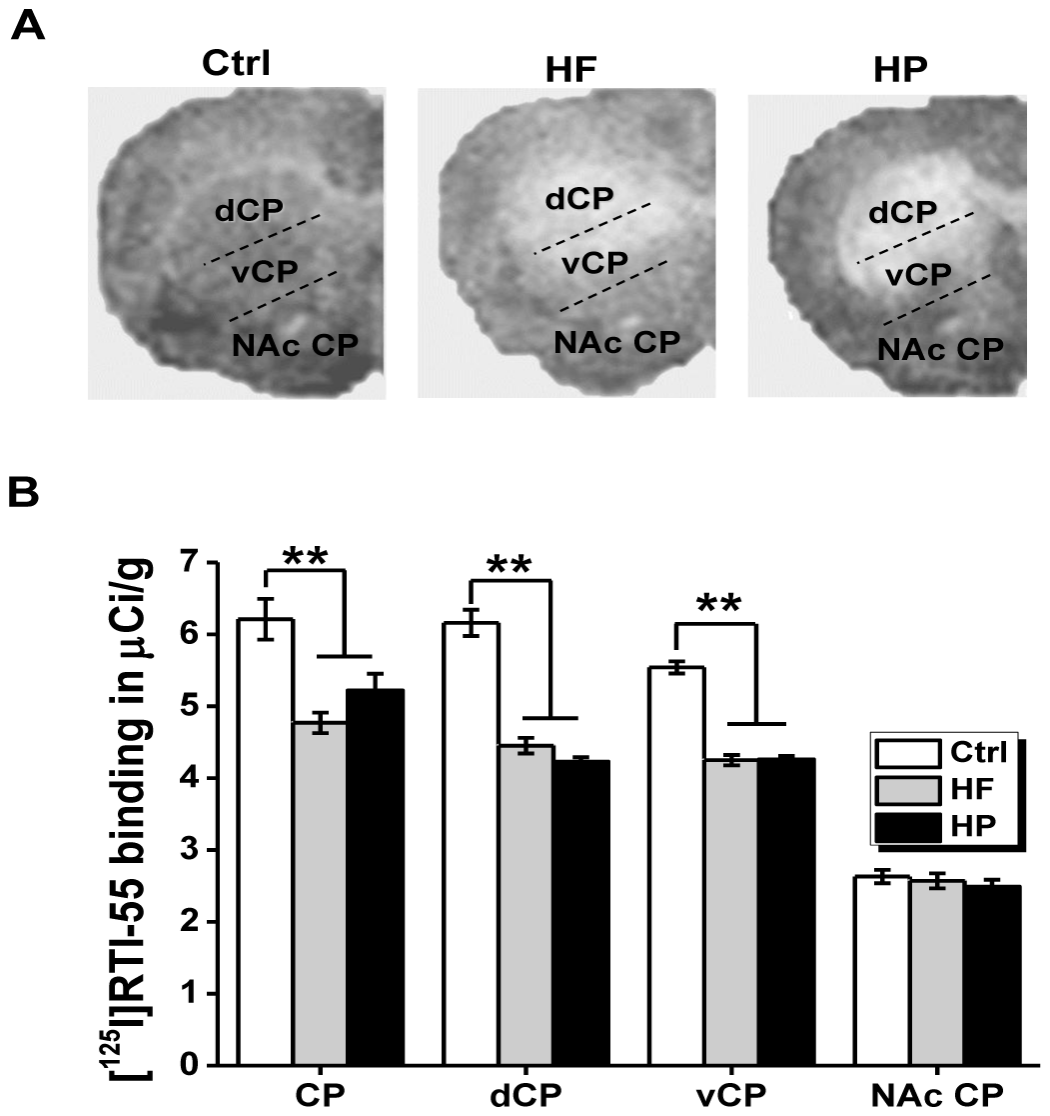
[125I]RTI-55 binding to striatal dopamine transporter (DAT). (A) Representative autoradiographs for each group for DAT binding in the striatum of Wistar rats fed with the control diet (Ctrl), high fatty (HF) or highly palatable (HP) diet. Several regions were observed: caudate-putamen (CP), the dorsal (dCP) and ventral (vCP) subdivisions of the CP, and the nucleus accumben (NAc). (B) Values for DAT binding in the striatum of Wistar rats fed with the control diet (Ctrl), high fatty (HF) or highly palatable (HP) diet (n = 9 for Ctrl group, n = 10 for HF group and n = 11 for HP group). Values were generated by quantitative autoradiography of ligand binding. **p<0.001, compared as indicated.

After behavioral tests, rats (n = 9 for Ctrl group, n = 10 for HF group, and n = 11 for HP group, Fig. 5A–B) were anesthetized with an overdose of sodium pentobarbital (250 mg/kg, i.p.), decapitated, and their brains were removed and frozen at −20°C by immersion in isopentane. A measure of 20 µm-thick coronal sections were cut on a cryostat at the level of the anterior striatum (AP coordinates +1.7 mm to +0.8 mm, according to Paxinos and Watson [19]), thaw-mounted on Vectabond-treated glass slides and stored at −20°C for autoradiography use. Warmed slides removed from the −20°C freezer were preincubated in a solution of assay buffer (10 mM Na_3_PO_4_, 120 mM NaCl, 100 mM sucrose) containing 100 nM fluoxetine for 5 min to remove endogenous ligands that could interfere with subsequent radioactive ligand binding. As [^125^I]RTI-55 bound with high affinity to both DAT and serotonin transporter (SERT), 100 nM fluoxetine was included in both the preincubation and incubation media to block the radioligand binding to SERT. After preincubation, the sections were incubated in a solution of assay buffer containing 25 pM [^125^I]RTI-55 and 100 nM fluoxetine for 2 h. The sections were then rinsed twice for 2 min each at 41°C in assay buffer, then once for 10 s in 41°C distilled water. The rinsed slides were then rapidly dried under a stream of heated air. The dried slides and [^14^C]-containing autoradiographic standards were opposed to Hyperfilm MP (GE Healthcare) for 48 h before development. Quantification of [^125^I]RTI-55 binding was done using a medical image analyzer (Shenteng Ltd, Shanghai, China). Image densities were converted to [^125^I]RTI-55 binding levels using a calibration curve based on images of the standard slides packed with each film. Regional densities of RTI binding were obtained by outlining the desired structures on their respective [^125^I]RTI-55 images. Values obtained represented the average of measurements taken from both hemispheres in a total of four sections per animal. For analysis, the image of striatum (see Fig. 5A) was first subdivided into caudate-putamen (CP) and nucleus accumbens septi (NAc). The CP was then subdivided into dorsal (dCP) and ventral (vCP) parts, which were separately quantified for [125I]RTI-55 binding.

### Experiment 8: Western blot analysis

**Figure 6:**
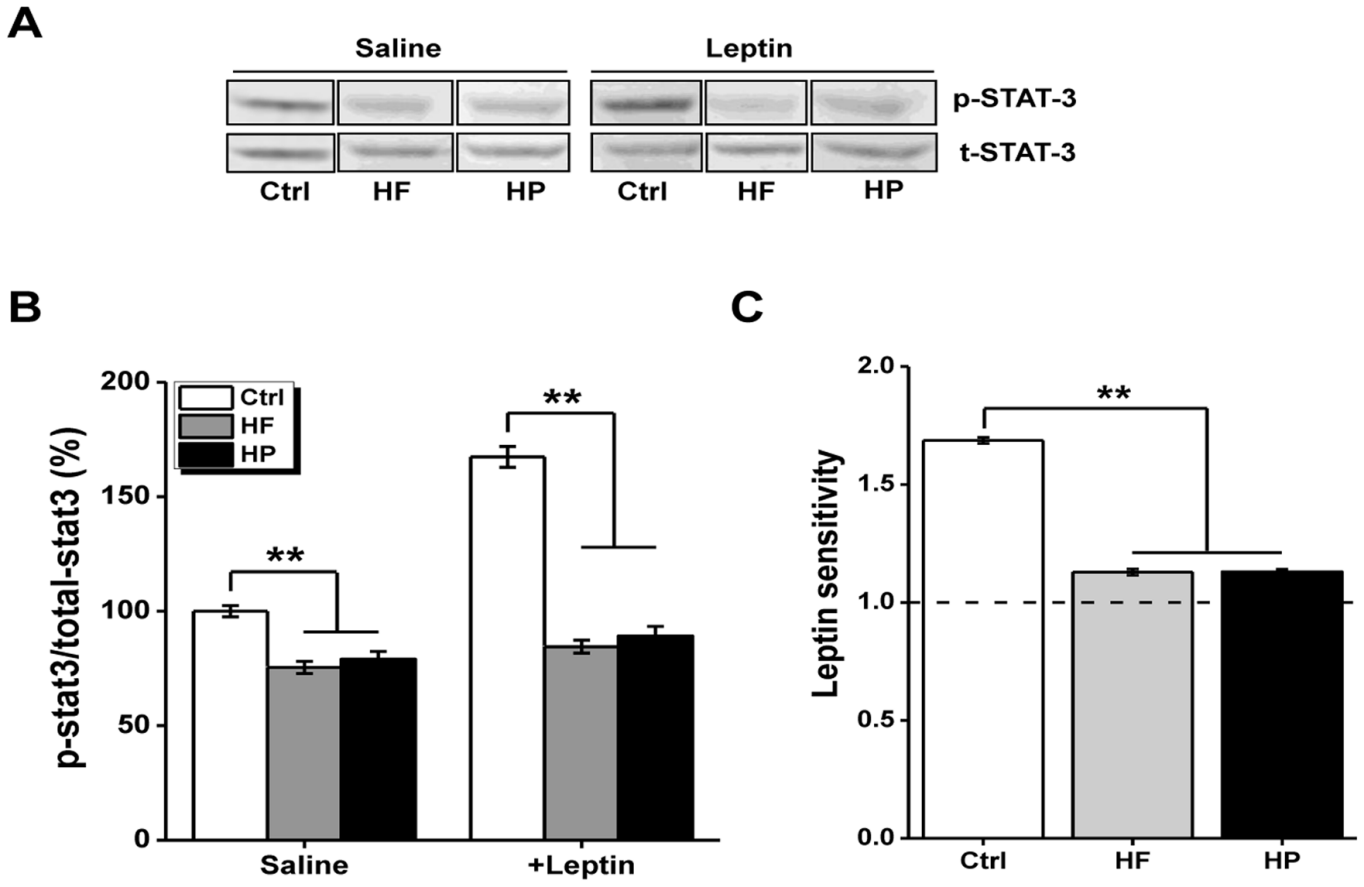
Phosphorylation of STAT-3 in the hypothalamus. (A) Representative images for western blot experiement on total (t-) and phosphorylated (p-) STAT-3. n = 9 for Ctrl group, n = 10 for HF group, and n = 11 for HP group, respectively. (B) Measurement of the p-STAT-3/total-STAT-3 ratio in saline injected rats and in leptin injected rats. For clearly comparison, all the p-STAT-3/t-STAT-3 ratios were normalized to the mean value that obtained from the Ctrl group. (C) The sensitivity toward leptin assessed by a significant elevation of the mean p-STAT-3/t-STAT-3 in leptin-injected compared to saline-injected rats. **p<0.001, compared as indicated.

Samples were prepared as previously described [20]. Briefly, frozen hypothalami were homogenized in lysis buffer: 10 mM Tris-HCl (pH 7.5), 150 mM NaCl, 1 mM EGTA, 1mM EDTA, 0.5% nonidet-P40, 1% Triton X-100, protease inhibitor cocktail (0.35 mg/ml PMSF, 2 µg/ml leupeptin and 2 µg/ml aprotinin), and phosphatase inhibitor cocktail (10 mM sodium fluoride, 1 mM sodium orthovanadate, 20 mM sodium β-glycerophosphate, and 10 mM benzamidine). After lysis in ice for 90 min, insoluble materials were removed by centrifugation (15,000 rpm at 4°C for 45 min), and protein concentrations of the resulting lysates were determined using a protein assay kit (Bio-Rad Laboratories, Hercules, CA, USA). Proteins (50 µg) were subjected to SDS-PAGE and transferred onto nitrocellulose membranes. Blots were blocked with 5% nonfat milk and then incubated in the presence of appropriate primary antibodies (anti-phosphorylated STAT-3 or anti-total STAT-3 from Cell Signaling Technology; Danvers, MA, USA) and secondary antibodies. Following nitrocellulose membrane washing, targeted proteins (about 92 kDa) were revealed using enhanced chemiluminescence reagents (Super-Signal, West Femto, Pierce, Rockford, IL, USA). The intensity of bands was quantified by using medical image analysis software 2.0 (Shenteng Information Technology, Shanghai, China) (Fig. 6A). In each group, the phosphorylated STAT-3 (p-STAT-3) levels were normalized to total STAT-3. The p-STAT-3/total-STAT-3 ratio was measured in saline injected rats and in leptin injected rats. For clearly comparison, all the p-STAT-3/t-STAT-3 ratios were normalized to the mean value that obtained from the Ctrl group (Fig. 6B). The sensitivity towards leptin was defined as an elevation of mean p-STAT-3/t-STAT-3 ratio in leptin-injected compared to saline-injected rats (Fig. 6C).

### Statistics

Data were analyzed using the SAS software package (Version 11.8). One-way ANOVA or repeated measures ANOVA were applied to detect intergroup differences. If significant interactions were found, they were further explored through post hoc comparisons (Newman–Keuls) to establish simple effects. Values were expressed as the mean ± S.E.M., *P<0.05, and **P<0.001 was considered statistically significant.

## Results

### Effect of maternal diet on dam, baby and adult offspring

In order to confirm that the overnutrition-induced maternal obese models were successfully set up, body weight of dam was measured at the following time points. At 6 weeks before mating, no difference was found for dam body weight among HF and HP diet groups as well as their counterparts (Fig. 1A, F _(2, 27)_ = 0.03, P = 0.97); After HF and HP diet, the dam body weight of both HF and HP groups were higher than Ctrl group at the time of mating (Fig. 1A, F _(2, 27)_ = 3.97, P = 0.03) and after the delivery of their offspring (Fig. 1A, F _(2, 27)_ = 6.82, P = 0.004), indicating that maternal obese model was successfully set up. Next whether maternal obese during pregnancy affected the basal physiological parameter of offspring was investigated. Weight of the pups at birth (Fig. 1B, F _(2, 27)_ = 0.14, P = 0.87) and the age of 10 weeks (Fig. 1C, F _(2, 27)_ = 3.047, P = 0.07) was similar among three different groups. Serum parameters, measured in three groups of adult offspring aged 10 weeks after overnight food deprivation, were showed in Fig. 1D–F. The offspring born to dams under either HP or HF diet displayed comparable levels of serum glucose (F _(2, 27)_ = 2.20, P = 0.13), insulin (F _(2, 27)_ = 2.64, P = 0.08) and leptin (F _(2, 27)_ = 0.76, P = 0.47) to those of control offspring, indicating basic physiological parameters were not significantly affected by the maternal obesity and prenatal overnutrition.

### Visual discrimination and serial reversal learning

In visual discrimination task (Fig. 2A), three groups of offspring rats did not differ in the number of trials (Fig. 2B, F _(2, 27)_ = 1.86, P = 0.18) and errors (Fig. 2C, F _(2, 27)_ = 0.42, P = 0.66) to reach the criterion on the acquisition of instrumental spatial discrimination. Meanwhile, no performance difference in the number of trials (Fig. 2B, F _(2, 27)_ = 0.09, P = 0.91) or errors (Fig. 2C, F _(2, 27)_ = 0.30, P = 0.75) to criterion on the retention was observed in the groups, which indicated three groups rats had comparable capability in the discrimination visual discrimination.

Next we investigated the reversal learning of offspring. A repeated measures ANOVA was conducted for testing the differences in the number of trials or errors to criterion, which revealed significant differences in main effects of maternal diet on the number of trials or errors to criterion (Fig. 2B, F _(2, 27)_ = 18.09, P<0.001 for trials; Fig. 2C, F _(2, 27)_ = 38.74, P<0.001 for errors) among three groups, but no significant difference between maternal HF and HP diets (P>0.05). We found significant interaction of maternal diet × reversal in trials (F _(4, 54)_ = 3.65, P<0.001), of maternal diet × reversal in errors (F _(4, 54)_ = 5.51, P<0.001), and an obvious main effect of reversal (F _(2, 54)_ = 33.32, P<0.001 for trials; F _(2, 54)_ = 45.75, P<0.001 for errors), which indicated experience might enhance reversal performance in all three groups of rats. Besides, the performance deficits (also indicated by more trials and errors to criterion) caused by maternal diets were selectively observed in the early phase of reversals (Rev1) (Fig. 2B, F _(2, 27)_ = 34.29, P<0.001 for trials; Fig. 2C, F _(2, 27)_ = 69.6, P<0.001 for errors), but not in the middle or late phase of reversals (Rev2 or 3) (F _(2, 27)_ = 0.97, P = 0.39 and F _(2, 27)_ = 0.19, P = 0.82 for trials respectively in Rev2 and Rev3, Fig. 2B; F _(2, 27)_ = 3.04, P = 0.06 and F _(2, 27)_ = 0.13, P = 0.88, for errors in Rev2 and Rev3, respectively, Fig. 2C).

Between reversal learning phases, retention of reversal learning was compared. Rats in three groups didn’t show any difference to retain the reversals. A repeated measures ANOVA analysis revealed there were no significant main effect of maternal diet (Fig. 2B, F _(2, 27)_ = 1.00, P = 0.38 for trials; Fig. 2C, F _(2, 27)_ = 1.20, P = 0.32 for errors), retention phase (Fig. 2B, F _(2, 54)_ = 0.01, P = 0.92 for trials; Fig. 2C, F _(2, 54)_ = 2.32, P = 0.14 for errors) and interaction (Fig. 2B, F _(4, 54)_ = 0.10, P = 0.91 for trials; Fig. 2C, F _(4, 54)_ = 0.44, P = 0.65 for errors).

When we further explored the type of errors (perseverative and learning) as described in the methodology, we found there were significant difference in main effects of maternal diet (F _(2, 27)_ = 52.52, P<0.001), reversal (F _(2, 54)_ = 1002.45, P<0.001) and interaction (F _(4, 54)_ = 15.89, P<0.001) for the number of perseverative errors across all reversals (Fig. 2D), but no significant differences in main effects of maternal diet (F _(2, 27)_ = 0.58, P = 0.57), reversal (F _(2, 54)_ = 0.41, P = 0.53) and interaction (F _(4, 54)_ = 0.09, P = 0.92) for the number of learning errors (Fig. 2D). Besides, the difference in perseverative errors caused by maternal diets didn’t display in Rev2 (F _(2, 27)_ = 1.38, P = 0.27) and Rev3 (F _(2, 27)_ = 0.25, P = 0.78) but exclusively in Rev1 (F _(2, 27)_ = 99.92, P<0.001) (Fig. 2D), where post hoc analysis revealed significant difference between Ctrl and the other groups (P<0.001), but not within HF and HP group (P = 0.13) (Fig. 2D).

Additional analyses were undertaken to investigate whether maternal obesity caused by different diet led to any reversal differences in response to positive and negative feedback. Fig. 2E displayed the probability of shifting after an error or a correct response in three groups across all reversals. Two way ANOVA analysis of arcsine-transformed probability data from three groups of rats revealed a significant main effect of maternal diet (F _(2, 54)_ = 44.06, P<0.001) and interaction in maternal diet × feedback (F _(2, 54)_ = 47.54, P<0.001), collapsed across all three reversals. Simple main effects of maternal diet for each feedback type revealed maternal diet caused no difference in the probability of shifting after an correct response (shift given reward, F _(2, 27)_ = 1.22, P = 0.31), but significant difference in the probability of shifting after an incorrect response (shift given no reward, F _(2, 27)_ = 67.99, P<0.001), consistent with their marked perseverative responding to the previously rewarded, but now incorrect stimulus. Subsequent post hoc analysis of the “shift given no reward” data revealed no significant difference between HF and HP groups (P = 0.92).

As shown in Fig. 3A, during the discrimination or reversal phases there were no any differences between groups in the latency to collect the food reward (P value more than 0.05 for all comparisons). Besides, there were no differences on the amount of food consumed by offspring born to dam fed on different diet in a 30 min test where pellets were freely available (Fig. 3B, F _(2, 27)_ = 0.96, P = 0.40). Both HP and HF groups showed similar levels of spontaneous locomotor activity (ambulatory movement) as control subjects, as measured by the number of beam breaks during the 60-min test session (Fig. 3C, F _(2, 27)_ = 0.68, P = 0.51). These data indicated again that three group rats had similar performance in most behaviors.

### Attentional Set-Shifting Task

During training, the rats readily learned to discriminate food-baited bowls based on either scents or media (Fig. 4A). Testing was conducted over seven phases as shown in Fig. 4A. A repeated measure ANOVA was conducted to investigate the differences in trials-to-criteria and errors made for three groups. For the trials-to-criterion measure, the main effects of maternal diet (F _(2, 27)_ = 58.80, P<0.001), phase (F _(6, 162)_ = 16.36, P<0.001) and the phase × maternal diet interaction (F _(12, 162)_ = 10.92, P<0.001) were significant across all the phases (Fig. 4B). For the errors measure, the effects of maternal diet (F _(2, 27)_ = 46.98, P<0.001) and phase (F _(6, 162)_ = 51.32, P<0.001) were significant but not interaction (F _(12, 162)_ = 0.06, P = 0.94) (Fig. 4C). Subsequent post hoc tests revealed that HF group and HP group required more trials-to-criterion and made more errors than did Ctrl group for the phases of CDr (F _(2, 27)_ = 66.87, P<0.001 for trials, Fig. 4B; F _(2, 27)_ = 21.90, P<0.001 for errors, Fig. 4C), IDr (F _(2, 27)_ = 38.06, P<0.001 for trials, Fig. 4B; F _(2, 27)_ = 39.47, P<0.001 for errors, Fig. 4C) and EDr (F _(2, 27)_ = 21.03, P<0.001 for trials, Fig. 4B; F _(2, 27)_ = 33.95, P<0.001 for errors, Fig. 4C) but not for the other four phases (P values >0.05 for all comparisons).

However, when comparing the effect of maternal diet on the attentional set-shifting of offspring, indicated by ED-ID difference scores, no differences were detected in 3 groups (F _(2, 27)_ = 0.27, P = 0.77 for trials-to-criterion Fig. 4B; F _(2, 27)_ = 0.90, P = 0.42 for errors, Fig. 4C). As shown in Fig. 4B–C, our findings showed that all the offspring rats in 3 groups required more trials to reach criteria, and committed more errors for the discrimination requiring an extradimentional shift, comparing with intradimentional shift (paired sample t-tests. P values <0.001 for trials-to-criterion and errors). But, the ED-ID difference scores didn’t significantly differ in 3 groups, indicating that maternal obesity induced by overnutrition didn’t impact the attentional set-shifting abilities of offspring.

### Altered dopamine homeostasis upon maternal obesity

In order to explore the potential molecular mechanisms, we investigated whether expression of dopamine (DA) transporter (DAT) in striatum might be influenced by the maternal obesity due to overnutrition, as imbalanced striatal DA homeostasis was reported to be associated with reversal learning deficit, especially in early phase [21]. In Fig. 5A–B, analysis of [^125^I]RTI-55 binding to DAT revealed that maternal HF and HP diets produced a significant reduction of DAT within CP (F _(2, 27)_ = 14.23, P<0.001) of their offspring, whereas NAc DAT was unaffected (F _(2, 27)_ = 0.51, P = 0.60). Further analysis revealed that this depletion of DAT occurred in CP of both dCP (F _(2, 27)_ = 68.97, P<0.001) and vCP (F _(2, 27)_ = 114.94, P<0.001). However, no regional DAT binding difference was found within HF and HP group (P = 0.23).

### Leptin-dependent STAT-3 phosphorylation in the hypothalamus

To gain additional mechanistic insights on the cause of altered striatal DA homeostasis upon maternal overnutrition, we have turn to examine the consequent changes of central leptin signaling, a pathway frequently affected in obesity. The hypothalamus, especially the lateral hypothalamic area acts in concert with the ventral tegmental area and other components of the mesolimbic DA system to shape diverse aspects of animal behaviors [22], [23]. Mechanistically, the defected leptin signaling in hypothalamus associated with obesity probably confers partially at least for the altered striatal DA homeostasis upon maternal overnutrition. To assess the central leptin signaling in hypothalamus, the phosphorylation levels of STAT-3 under basal conditions and in response to a bolus of leptin in three groups of the offsprings were compared. Ten week-old rats of three groups were starved overnight and divided into three groups that received saline or leptin (i.p.), respectively. In each group, STAT-3 phosphorylation levels were normalized to total STAT-3. Under basal conditions, the levels of pSTAT3 in both HF and HP groups were significantly lower compared to control group (Fig. 6A–B), implying the disrupted leptin signaling upon maternal obesity, since the circulating levels of leptin in the offspring born to dams under either HP or HF were comparable to that from control offsprings (Fig. 1F). Similarly, under the condition in response to leptin, the levels of pSTAT3 in these groups (i.e. HF and HP) were also substantially reduced (Fig. 6A–B). Moreover, the quantification of leptin sensitivity by assessment of the elevation of p-STAT-3/t-STAT-3 ratio in leptin-injected compared to saline-injected rats (Fig. 6C) showed that the significant increase (∼68%) of STAT-3 phosphorylation observed in the adult offspring born to normally fed dams was totally abolished in those born to HF or HP dams (F _(2, 27)_ = 68.46, P<0.001), while no significant group difference was found between HF and HP group when post hoc analysis was further explored (P = 0.58) (Fig. 6A–C). Together, a defected central leptin signaling indeed occurred in offspring that exposed maternal obesity caused by overnutrition, which probably underlies the aforementioned deficiency in reversal learning and striatal DA disturbance.

## Discussion

Maternal obesity is an increasing public health concern in developed countries and the developing world. In the United Kingdom, about 20% women of childbearing age are obese; in the United States this figure is up to 34% [20]. Epidemiological studies reveal that maternal obesity is associated with adverse outcomes for not only mothers but also children [24]. Neonates born to obese mothers are posited at higher risk of congenital anomalies, fetal macrosomia, and neonatal unit admission [24], [25]. Long-term consequences for these offspring include an increased risk of obesity in their later life [24], [26]. Besides, recent human studies have revealed that, in addition to obesity, type 2 diabetes, insulin resistance, and hyperleptinemia are also associated with pregnant obesity, which usually causes developmental programming through poor bio-communication between mother and fetus [27].

Furthermore, maternal obesity during pregnancy is recently reported to greatly affect central nervous system of offspring as well since the adverse neurodevelopmental outcomes such as low IQ performances and core symptoms of ADHD are observed in the children born to obese mothers [28], [29]. Corresponding to the report on the human investigation, animal studies have also suggested that maternal obesity indeed negatively affect the development and function of central nervous system in offspring [4]-[6]. For example, it was found that maternal obesity impaired spatial learning performance in the mouse offspring through the disruption of production of brain-derived neurotrophic factor (BDNF) in hippocampus [5]. However, further understanding of other behavioral impacts and related mechanisms are restricted. For example, both unfavorable nutritional status and poor physical activity during pregnancy may cause maternal obesity and subsequent developmental disorders in offspring through some shared or individualized mechanisms, which are not elucidated. Besides, it has reported that different dietary macronutrient intake, such as high fat diet or sucrose-rich diet, during pregnancy may play the incomparable influence on offspring [30], [31], indicating further studies are required. Moreover, current human studies can’t exclude the possibilities that the observed cognitive disadvantages might be a result of predisposed obesity developing in adult offspring, since offspring born to obese dams are more vulnerable to adulthood obesity which usually causes a disturbance in central neuronal system through defective insulin regulation. Thus, further investigation under appropriate body weight control of offspring need to be conducted.

In the present study, we found out for the first time that the rats born to obese dams were greatly impaired in reversal learning, as assessed by both visual discrimination reversal learning (VDSRL) and attentional set-shifting (ASST), however no significant abnormal serum metabolic parameters were detected simultaneously. The effect on VDSRL was highly specific, as it was contained within the early stage of reversal, particularly when animals committed more perseverative errors of shifting after given no reward rather than reward. With repeated practice offspring born to obese dams recovered from the impairments and reached criterion at a comparable rate with control rats in the late stage of reversal. Notably, there were no differences in acquisition and retention of discrimination phase as well as retention of spatial reversals across three groups, indicative of unaffected basal ability of learning and memory. In consistent with the findings in VDSRL, the reversal deficit was also observed in ASST, where the rats born to obese dams displayed slower learning abilities during reversal CDr, IDr and EDr, while left the set-shifting ability intact in ID/ED shift (represented by ED-ID difference scores). Considering the increased errors found in the reversal learning paradigms possibly confounded by other behaviors, such as locomotor activity and food motivation, we conducted tests for measurement of ambulatory movements and latency to collect food pellets. Our findings indicated that all the offspring exhibited comparable food intake, body weight, as well as basal ambulatory movements, indicating performance deficits observed in the offspring born to obese dams indeed attributed to a failure to environmental adaptivity.

Additionally, these reversal impairments were found in offspring whose dams fed on HP diet or HF diet, and displayed no significant difference within two groups, indicating that the postnatal reversal abnormalities induced by maternal overnutrition are likely irrespective of whatever kind of diet intake during pregnancy. In order to verify whether the observed reversal deficits were dependent on their predisposed obesity in the later life, we fed the offspring on the standard chow after weaning and kept their body weight in appropriation control before the behavioral tests were finished. Our results revealed that disturbed reversal learning of offspring was indeed independent of their body weight, indicating maternal obesity during pregnancy was the main cause, instead of predisposed obesity in offspring.

Anatomically, reversal learning involves orbitofrontal cortex (OFC) and dorsal striatum [32]–[37]. In our studies, impairments in reversal learning strongly suggested that there might be a dysregulation of OFC-striatum pathway in offspring born to the obese dam. More specifically, the abnormal response was selectively found to a previously rewarded but now incorrect stimulus rather than a previously no-rewarded but now correct stimulus, arguing a more likely involvement of striatum rather than OFC, since striatum is particularly sensitive to negative outcomes while the OFC sensitive to both negative and positive outcomes [14]. At present, it has been long considered that OFC-striatum pathway subjects to neuromodulatory inputs by DA, serotonin (5-HT), and noradrenaline (NA) [32]–[37]. In line with that, we found reduced DA uptake function in both dorsal and ventral divisions of the CP, with ventral CP in a greater extent of deficiency, indicating compromised striatal circuitry caused by abnormal DA clearance might underlie the maladaptive responding reported above. Also, the present interpretation linking striatum DA disturbance to the specific reversal learning deficits accords well with a recent report that medial striatal lesions in monkeys produced perseverative impairments during reversal learning [14]. Furthermore, a recent microdialysis study demonstrated a specific role of DA in the early but not late stage of reversal learning [21], which fit well with the observed selective behavioral impairments in VDSRL that only occurred in the early but not late phases. Specially, the activated DA efflux has been demonstrated increased in rats performing the first reversal compared with controls performing the previously acquired discrimination, but was absent during a third reversal [21], supporting the particular involvement of DA activity for newly formed positive response-reward associations during reversal learning. By contrast, NA was considered to play less important role in reversal learning, as NA efflux was only weakly activated during most phases of reversal learning [21]. Recently, a group of studies suggested that improved reversal learning could be caused by increased NA efflux through administration of α2-receptor antagonist such as idazoxan [38] and atipamezole [39], supporting a part for NA in cognitive flexibility. However, this conclusion would be argued by the fact that all α2-receptor antagonists, including idazoxan and atipamezole, increase cortical DA efflux in addition to NA efflux [40], [41]. Collectively, we prefer to the view that the disturbed DA activity most likely underlie here identified cognitive rigidity upon maternal overnutrition, although the possible involvement of NA action are not absolutely excluded.

In an attempt to understand mechanisms underlying the abnormalities in striatal DA clearance and reversal learning caused by maternal obesity, we investigated the regulation of neuronal leptin signaling, a critical link between maternal environmental factors and the developing physiology of the infant. Functionally, neuronal leptin contributes to preventing the storage of excess adipose tissue by feedback to the hypothalamus to reduce food intake and increase energy expenditure [42]. However, in most human with obesity, system leptin levels are often responsively elevated due to the leptin resistance [43]. Recent researches reveal that leptin signaling is exquisitely sensitive to maternal diet during prenatal and early postnatal life, which possibly plays a regulatory influence on striatal DA function [27]. It had been reported that the highest levels of leptin expression were found in the hypothalamus. Specially, recent report indicated that in the young age, at least prior to about 8 week of age, hypothalamus was the principal site of leptin-mediated regulation, with increasing age, the functions of other neuronal networks gradually increased [44]. Thus, it was proposed that hypothalamic leptin signaling was critical for establishing essential baselines in young animals that are maintained through the actions of distinct signaling networks in mature animals. Considering all the offspring rats under our investigation were as young as about 10 weeks old, we assumed hypothalamus could probably remain as the key brain region for regulation of leptin signaling. Research from rodent models had illuminated the underpinnings of leptin's ability to modulate emotion and neurobehavioral responses through the modulation of DA by leptin receptors [23]. Anatomically, leptin receptors are located in many brain regions that influence dopaminergic neurotransmission including areas of DA synthesis and projection sites such as striatum and nucleus accumbens, and via leptin receptor expressing neurons of the lateral hypothalamus that modulate dopaminergic neurons in the ventral tegmental area. In that regards, the defected leptin signaling in hypothalamus upon maternal overnutrition probably confers in part at least for the altered striatal DA homeostasis in the offspring.

Depending on the anatomical target, central leptin signaling was considered to participate into regulating many DA-dependent behaviors [22], such as substance abuse, locomotor activity, enforced leaning, food intake, sleep and adaptive behaviors. Thus, it could be speculated reversal learning, as one of adaptive behaviors associated with DA activity might be potentially regulated by leptin signaling, which however needs further elucidation. In our studies, we examined final body weight, circulating leptin level and assessed the hypothalamic leptin sensitivity by measuring leptin-dependent STAT-3 phosphorylation. We found that offspring born to obese dams fed on HP or HF diet normally exhibited body weight and related plasma parameters like in serum leptin and insulin levels, were quite similar to those of control rats. However, among three groups of adult male rats born to HF, HP and control dams, only control group displayed an increased phosphorylation level of STAT-3 in the hypothalamus in response to leptin challenge, implying that offspring born to the obese dams brought an early defective leptin signaling, which might account for abnormal striatal DA modulation and consequent impaired adaptive behaviors observed in the former experiments. It was worthy to note that some other potential routs such as phosphoinositide 3-kinase-protein kinase B (Akt)-extracellular signal-regulated kinase (PI3K-Akt-ERK) pathway may also bear the defective leptin signaling, which warrant the further investigation. Considering that defective leptin signaling is also frequently found as a shared etiology in obese patients with physical inactivity, we might hypothesize that here identified reversal learning deficits caused by maternal overnutrition probably could be applied to pregnant obesity caused by physical inactivity as well, which needs further investigation in the future.

In summary, our data demonstrate that maternal obesity during pregnancy, caused by either HF or HP food, disrupts the reversal learning through an altered leptin signaling in offspring. Such disrupted leptin signaling may contribute to the disturbance in DA homeostasis in striatum and consequently cause above mentioned behavioral abnormalities. In line with other reports in human and animal studies, our findings add new level of evidence that maternal obesity cause the long-term impairments in cognitive flexibility. Our findings reinforce the importance of healthy weight achievement during pregnancy and provide cues for understanding the related mechanistic processes by which priming of risk for developmental disorders may occur during early life.
